# Varying degrees of spontaneous osteogenesis of Masquelet’s induced membrane: experimental and clinical observations

**DOI:** 10.1186/s12891-023-06498-4

**Published:** 2023-05-15

**Authors:** Qudong Yin, Xueming Chen, Beichen Dai, Jun Liu, Ying Yang, Sheng Song, Yanping Ding

**Affiliations:** 1 Department of Orthopaedics, Wuxi Ninth People’s Hospital Affiliated to Soochow University, Wuxi, Jiangsu 214062 China; 2 Department of Radiology, Wuxi Ninth People’s Hospital Affiliated to Soochow University, Wuxi, Jiangsu 214062 China

**Keywords:** Bone defect, Induced membrane, Spontaneous osteogenesis, Osteogenic activity, Polymethylmethacrylate

## Abstract

**Background:**

Masquelet’s induced membrane (IM) has osteogenesis activity, but IM spontaneous osteogenesis (SO) has not been described previously.

**Objectives:**

To report on varying degrees of IMSO and analyze its possible causes.

**Methods:**

Twelve eight-week-old male Sprague-Dawley rats with 10 mm right femoral bone defects who received the first stage of IM technique (IMT) were used to observe the SO. In addition, clinical data from patients with bone defects who received the first stage of IMT with an interval of > 2 months post-operatively and exhibited SO between January 2012 and June 2020 were retrospectively analyzed. The SO was divided into four grades according to the amount and characteristics of the new bone formation.

**Results:**

At twelve weeks, grade II SO was observed in all rats, and more new bone was formed in the IM near the bone end forming an uneven margin. Histology revealed bone and cartilage foci in the new bone. Four of the 98 patients treated with the first stage of IMT exhibited IMSO, including one female and three males with a median age of 40.5 years (range 29–52 years). The bone defects were caused by severe fractures and infection in two cases and by infection or tumor in one case each. Partial or segmental defects occurred in two cases. The time interval between inserting a cement spacer and diagnosis of SO ranged from six months to nine years. Two cases were grade I, and one case each of grades III and IV.

**Conclusion:**

Varying degrees of SO confirm the existence of the IMSO phenomenon. Bioactive bone tissue or local inflammation and a long time interval are the primary reasons underlying enhancement of the osteogenic activity of IM and leading to SO, which tends to take place as endochondral osteogenesis.

## Introduction

Managing large segmental bone defects caused by trauma, osteomyelitis, and tumors is challenging, and many controversies exist concerning the optimal reconstruction method [1.2]. The three most reported surgical techniques are free vascularized fibular graft, Ilizarov bone transport and the Masquelet induced membrane technique (IMT) [1]. Each technique has advantages and disadvantages. The free vascularized fibular graft has a high union rate but requires excellent microsurgical experience and alters the donor’s healthy limb. The Ilizarov bone transport technique avoids a large amount of autogenous bone graft but requires a slow and painful repair process with a greater incidence of complications such as nonunion of the docking site and pin-path infection. The IMT is simple and easy to complete, but requires two stages. The first stage involves radical debridement and insertion of a cement spacer composed of polymethylmethacrylate (PMMA) into the bone defect. The second stage involves removing the spacer while leaving the induced membrane (IM) in place and filling the cavity with an autograft [2–5]. The IM has been shown to act in several ways. First, it serves as a protective physical barrier by preventing autograft resorption and second as a bioreactor by promoting healing through revascularization and growth factor secretion and concentrating mesenchymal stem cells (MSCs) with osteogenic properties. Therefore, the IM has osteogenic activity [6–10].

Membrane guided tissue regeneration (MGTR) was first proposed by Nyman in 1980s. In the study of periodontal tissue regeneration, it was found that a membrane was placed between the periodontal connective tissue flap and the root as a barrier, which can prevent the gingival junction level epithelium and connective tissue from growing into the periodontal, selectively guide cells with regenerative potential to proliferate on the root surface, and produce new cementum and periodontal ligament. Membrane guided bone regeneration (MGBR) technology was developed from the MGBR. It refers to a way to repair bone defects by placing membrane tube in the bone defect, creating a space conducive to bone growth, preventing non bone forming cells from invading into the bone defect, and collecting bone forming cells and growth factors. Then, the bone formation cells proliferate and differentiate to form new bone and grow along the membrane [11–13]. This technology emphasizes the importance of membrane in bone defect repair, but bone graft is still required for bone defect repair. However, several membrane materials have been found to small amount of new bone formation within the membrane in experimental studies of MGBR [11, 12].

MIT is a MGBR. We refer to the new bone formation without bone grafting at the bone defect as spontaneous osteogenesis (SO). In fact, in 2009, Klaue et al. [8] first noted new bone formation at the junction of the IM and bone end in sheep femoral defects receiving the first stage of IM technique at 16–18 weeks. However, they did not call the occurrence IMSO, and did not discuss the incidence, quantity, and features of the new bone formation. We observed varying degrees of IMSO in clinical practice and animal experiments. IMSO can shorten the treatment period of IMT and reduce the bone graft. To the best of our knowledge, no study has reported or investigated the varying degrees of IMSO. This study reported on the varying degrees of IMSO and analyzed its possible causes.

## Materials and methods

### Study design

This study using rats was reviewed and approved by the Institutional Animal Care and Use Committee of Wuxi No. 9 People’s Hospital (No. JY-KT20210132, registration date: October 10, 2021). All experiments were performed in accordance with the ethics laws and regulations of Soochow University and ARRIVE Guidelines for reporting animal research. This retrospective study on patients was reviewed and approved by the Review Board of Wuxi No. 9 People’s Hospital (No. JY-KT2021056, registration date: May 6, 2021) and performed in accordance with the ethical standards established in the 1964 Declaration of Helsinki and its later amendments. Written informed consents of participation in the study were obtained from the individuals (date: May 2021).

### Animal experiment

Twelve eight-week-old, specific pathogen-free, male Sprague-Dawley rats (mean weight 265 g, range 230–301 g) were used in this study. The rats were acclimated for one week before surgery to allow them to become familiarized with the new environment. The rats were kept in the animal experiment center (Soochow University Orthopedic Research Institute, Jiangsu, China). The rats were housed at a temperature of 20–23 ℃, a light cycle of 12 h of daylight/12 hours of dark, and the humidity of 60–80%. Sterile complete feed (Anlimo, Nanjing, China) and filtered water were freely available.

5% pentobarbital was injected intraperitoneally to induce general anesthesia. Aseptic techniques were used during the surgical procedures. The skin and fascia were cut longitudinally from the greater trochanter of the femur to the lateral condyle of the femur along the lateral side, creating an incision of about 2.5 cm. The subcutaneous muscles were separated to expose the lateral surface of the femur. Two osteotomies were created using a swing saw, and a 10 mm long, mid-diaphyseal bone segment was removed [5]. The bone defect was fixed with an intramedullary Kirschner wire and a prefabricated cylindrical antibiotic PMMA (2 g vancomycin/40 g) bone cement spacer (5 × 10 mm^2^). Finally, the muscles, the superficial fascia, and the skin were closed using absorbable sutures. The rats were treated with an antibiotic (cefuroxime sodium) for three days. The rats were allowed to return to full weight bearing immediately, post-operatively. All rats were euthanized with an overdose of pentobarbital administered intraperitoneally (150 mg/kg) at twelve weeks after surgery, routine disinfection was carried out, the rat was placed on sterile towels, and the leg was opened along the original surgical site. The cement spacer and intramedullary Kirschner wire were removed, and samples of the femur were taken for radiographic and histological examination.

### Patients

The inclusion criteria for patients were as follows: (i) bone defects were present in extremities without infection or with satisfactorily controlled infection before treatment with IMT in the Department of Orthopedics of Wuxi No. 9 People’s Hospital between January 2012 and June 2020; (ii) PMMA was used as a bone cement spacer in the first stage of the IMT procedure; (iii) radiographs, computed tomography (CT) or magnetic resonance imaging (MRI) and/or intraoperative exploration occurred after ≥ 2 months of insertion of the bone cement spacer, and (iv) varying degrees of SO were observed after the first stage procedure of IMT without bone grafting. The exclusion criteria were as follows: (i) patients had incomplete imaging and clinical data; (ii) local infection or recurrence of infection occurred after the surgical procedure; (iii) heterotopic ossification occurred; (iv) the patient presented with an ASA class III or above, and (v) the patient was receiving medication that might affect the bone remodeling procedure.

### Radiology equipment

1. Simens digital radiography system, Ysio Max.
2. GE Optima CT 660.
3. Simens Avanto DOT 1.5 T MRI.

### Spontaneous osteogenesis grading

According to the amount and characteristics of the new bone formation, the IMSO was divided into four grades. Grade (I) The IM became thick and hard, and bone precursor tissue (cartilage) formed at the bone defect, which was only detected by CT or MRI. Grade (II) Discontinuous new bone formation was observed on radiographs. Grade (III) Continuous new bone formation was observed only on one of the four sides of the bone defect using radiographs. Grade (IV) Continuous new bone formation was observed on two or more sides of the bone defect or accounted for 50% or more of the bone defect using radiographs.

Two senior attending physicians (one from the orthopedics department and one from the radiology department) were selected to grade the SO. When the two had different opinions, the grading was determined after discussion.

### Histological analysis

The specimens were fixed at 4 °C and soaked in decalcification solution (ethylenediaminetetraacetic acid 280 g, NaOH 30 g and 2 L PBS) for 4 weeks, dehydrated with alcohol, embedded in paraffin, sliced, and stained for histological analysis. We used Safranine O fast green stain: The sections were deparaffinized using conventional xylene, rehydrated to water, stained with iron hematoxylin for five minutes, differentiated with an acid differentiation solution for 15 s, then steamed and washed for ten minutes. Additional tissues were immersed in the green dye solution for five min, washed with a weak acid solution for ten s, dried, and sectioned. The sections were immersed in a safranine dye solution for five min, dehydrated in absolute alcohol for one min, and then sealed with a sealing agent.

## Results

### Experimental results

**Fig. 1:**
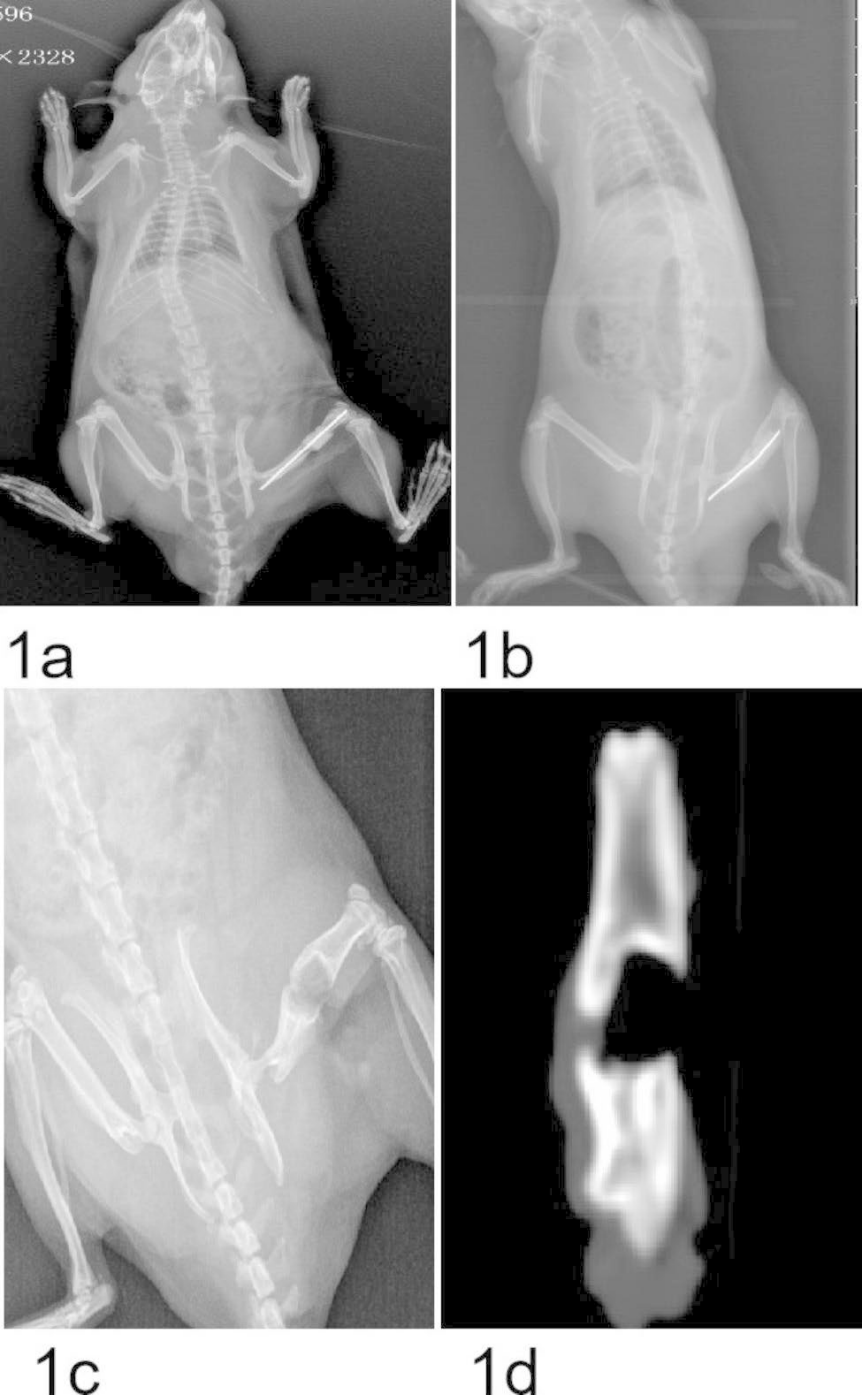
A male rat with a grade II IMSO. (**a**) Radiograph showing a cement spacer of PMMA inserted in the right femoral defect. (**b**) Radiograph showing discontinuous new bone formation at 12 weeks after insertion of the PMMA and thickening of the femur. (**c**) Radiograph showing discontinuous new bone formation after removal of the PMMA. (**d**) CT scans showing discontinuous new bone formation after the removal of the PMMA

**Fig. 2:**
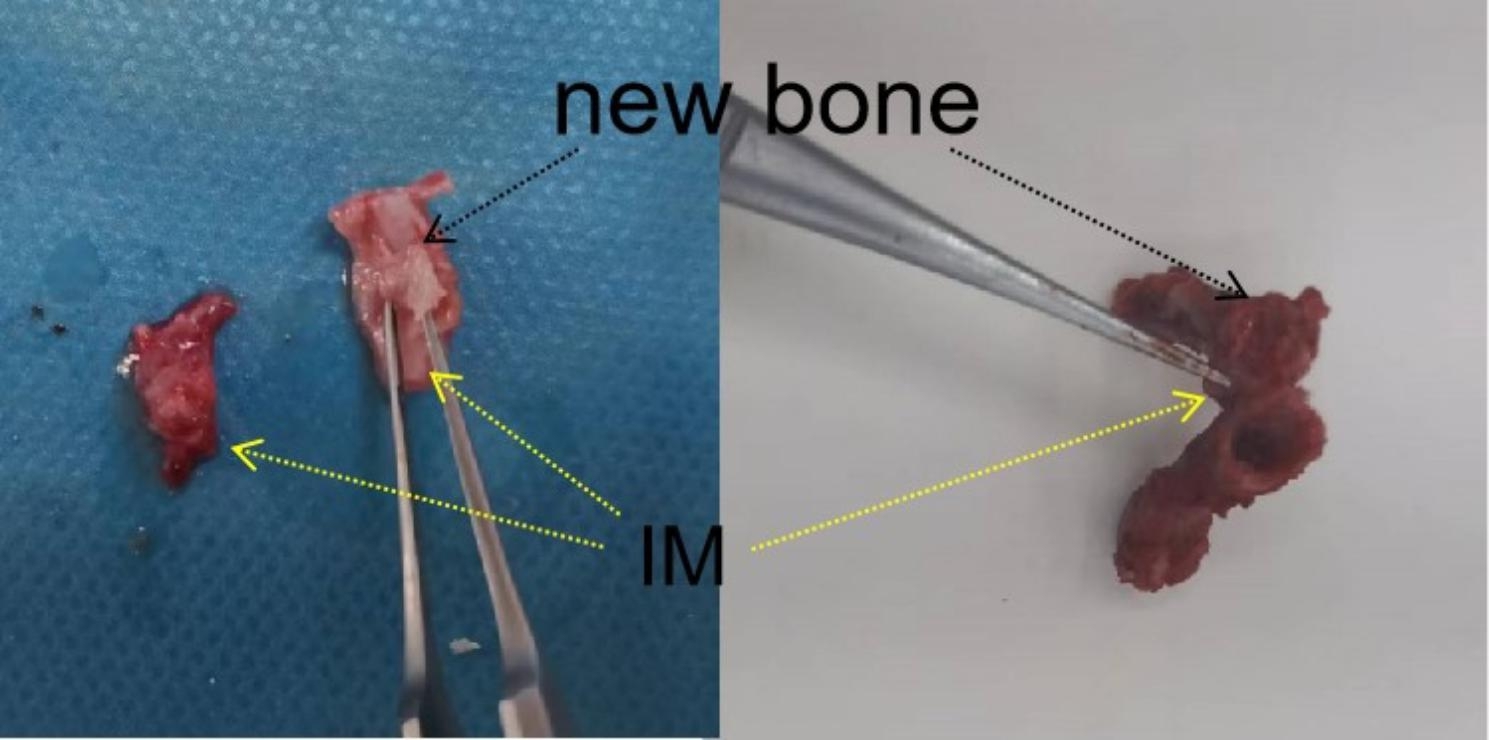
Gross morphological examination of the IMSO in an experimental rat. The IM was thickened and hardened, and new bone had formed

**Fig. 3:**
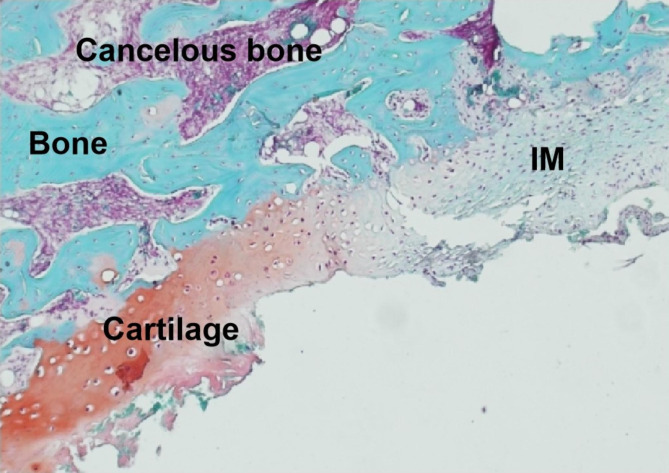
Histological examination of the IMSO in an experimental rat. Safranine O – fast green staining showed that in addition to the thickened IM (light blue), cancellous bone (purple), cartilage (red), and nature bone tissue (blue) were present

Grade II SO was observed in all twelve rats at twelve weeks. New bone formation occurred in the IM near the bone end and grew from the bone end to the center of the bone defects; an uneven lip was observed to wrap around the bone cement spacer with an average length of 3.1 mm (range, 2.0–6.0 mm; Figs. 1 and 2). Histological analysis showed that in addition to a thickened IM, cancellous bone, nature bone and cartilage were simultaneously appeared in newly formed bone within the IM (Fig. 3). Cartilage was located on the outer side while cancellous bone and nature bone were located on the inner side.

### Clinical results

**Fig. 4:**
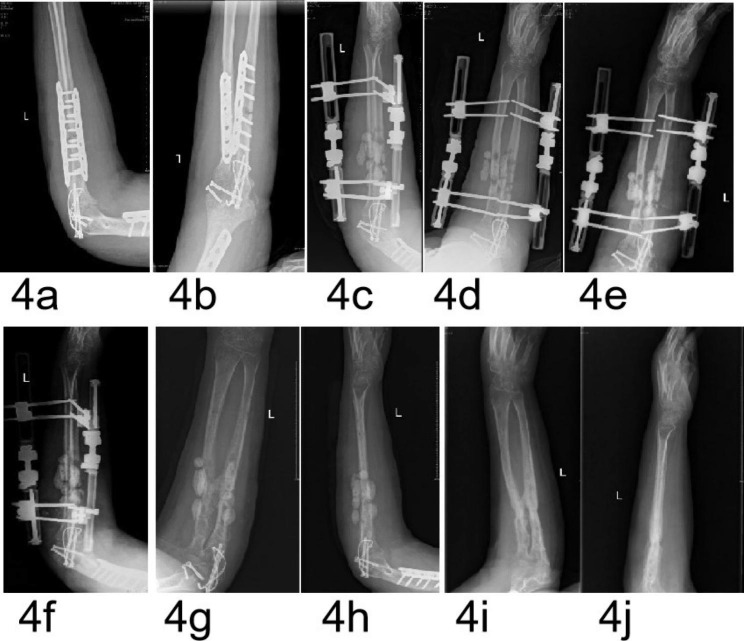
A 44-year-old female patient with a grade IV IMSO. (**a**,**b**) AP and lateral radiographs showing bone resorption caused by infection after internal fixation of ulnar and radial fractures. (**c**,**d**) AP and lateral radiographs showing the cement beads of PMMA inserted in the defects after debridement. (**e**,**f**) AP and lateral radiographs showing minimal new bone formation at three months after insertion of the PMMA. (**g**,**h**) AP and lateral radiographs showing continuous new bone formation at four months after insertion of the PMMA. (**i**,**j**) AP and lateral radiographs showing clinical healing of the fractures and defects at six months

**Fig. 5:**
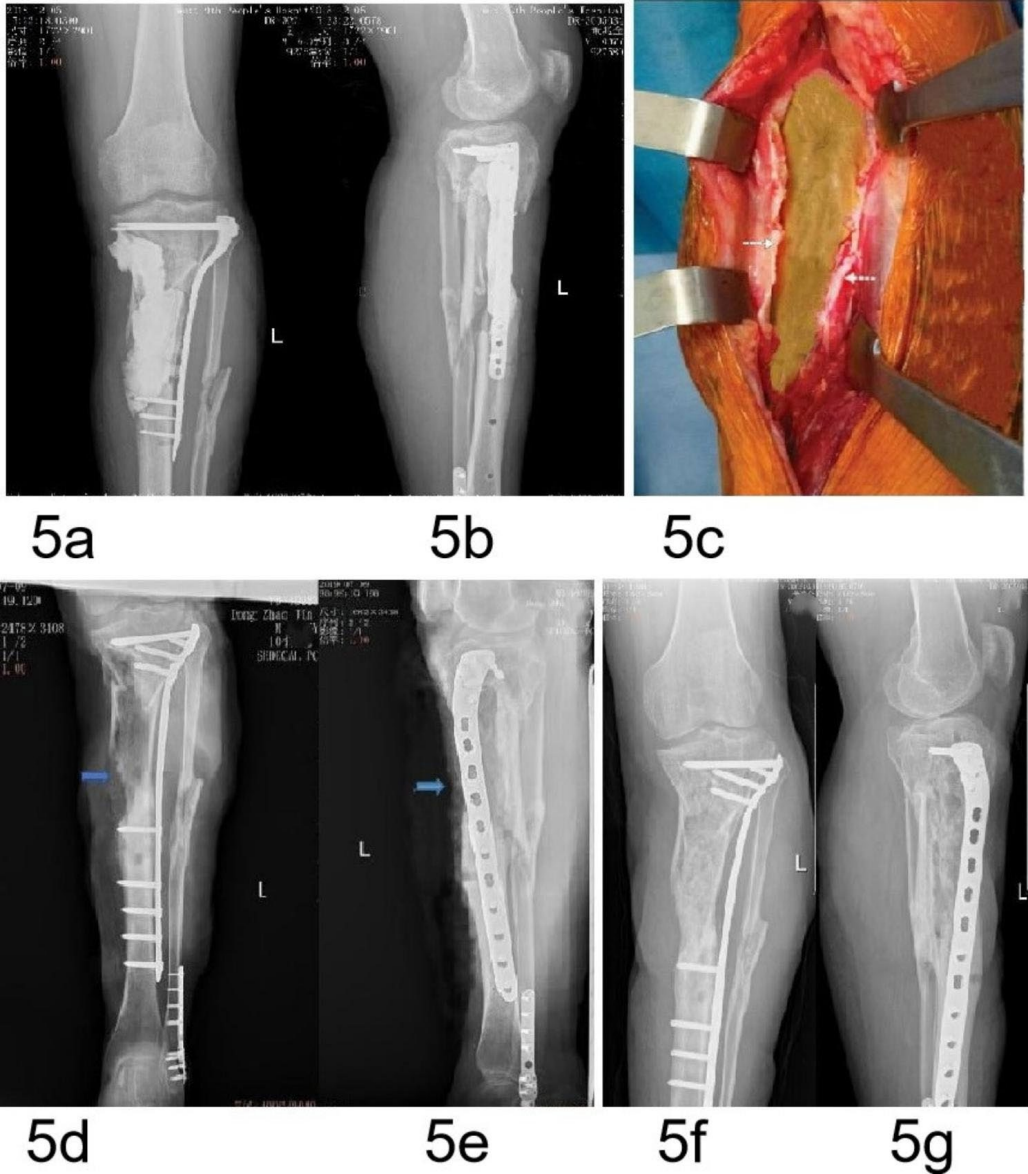
A 37-year-old male patient with a grade I IMSO. (**a**,**b**) AP and lateral radiographs showing that the cement spacer of the PMMA filled in the defects after debridement for infection of upper open tibial fractures. (**c**) Intraoperative exploration revealing thickening (2–3 mm) and hardening of the IM (white arrow). (**d**,**e**) AP and lateral radiographs showing indistinct new bone formation at nine months after insertion of the PMMA. (**f**,**g**) AP and lateral radiographs showing clinical healing of the fractures and defects after bone grafting

**Fig. 6:**
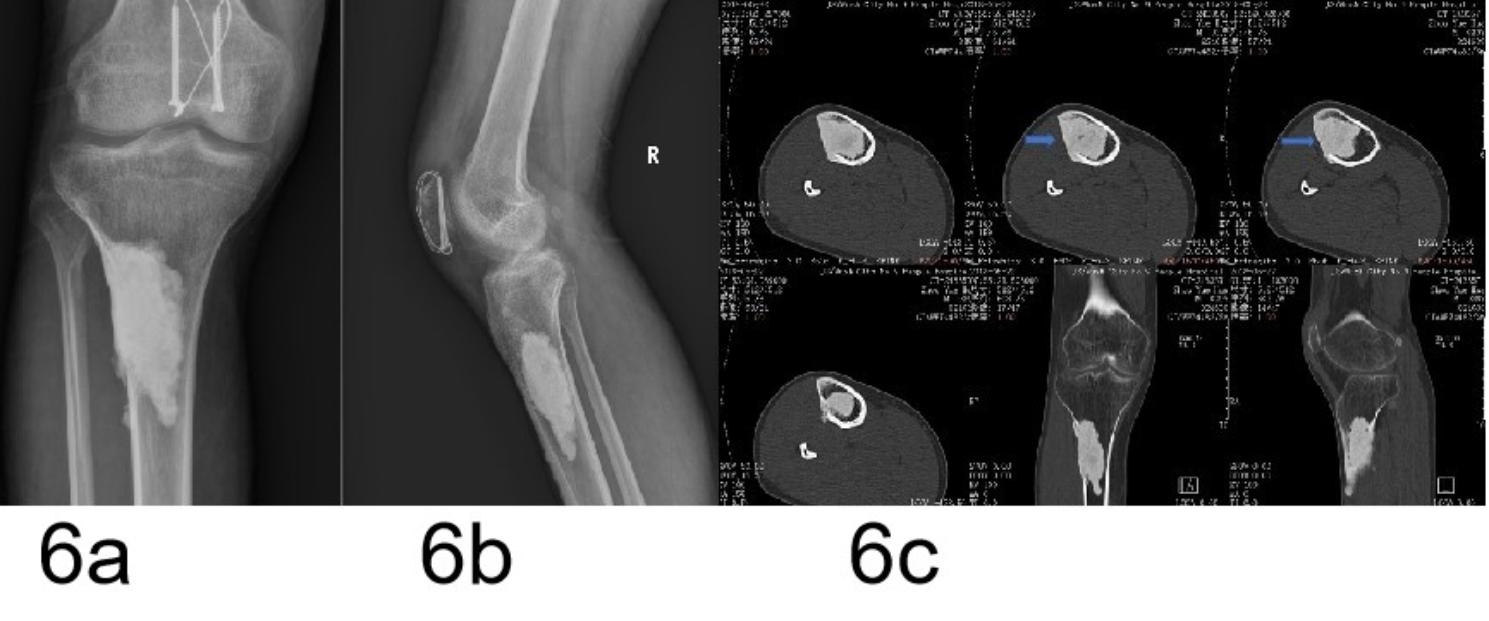
A 52-year-old male patient with a grade III IMSO. (**A**,**b**) AP and lateral radiographs showing cement spacer of PMMA inserted in the defects after resection for an upper tibial benign tumor. (**c**) CT scans showing continuous new bone formation (arrow) at nine years after insertion of the PMMA

**Fig. 7:**
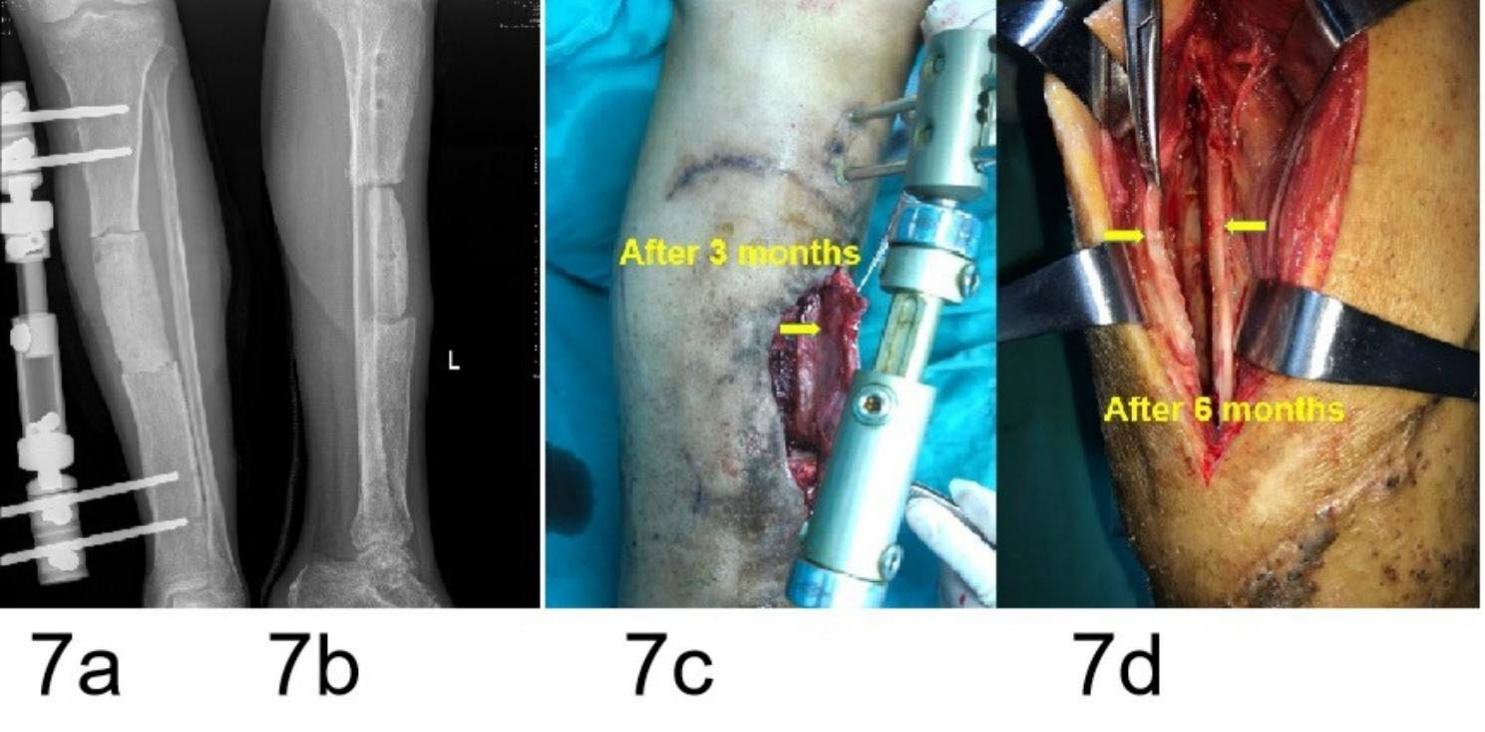
A 29-year-old male patient with a grade I IMSO. (**a**,**b**) AP and lateral radiographs showing the cement spacer of PMMA inserted in the tibial defects after resection due to osteomyelitis. (**c**) Intraoperative exploration revealing the IM (yellow arrow) was thin (1 mm) at three months after insertion of the cement spacer. (**d**) Intraoperative exploration revealing the IM was thicker (2–3 mm) and harder (yellow arrow) at six months compared to three months

Ninety-eight patients who underwent the first stage of IMT and performed radiographs, computed tomography (CT) or magnetic resonance imaging (MRI), and/or intraoperative exploration with an interval of > 2 months post-operatively were screened. Four patients (4.1%) presented with IMSO, including one female and three males with a median age of 40.5 years (range 29–52 years). The causes of the bone defects included severe fractures and infection in two cases, one case of infection, and one case with a tumor. The defects were in the tibia in three cases and the radius and ulna in one case. The median defect length was 6.3 cm (range 4.5–8.5 cm). The defects included two cases each of partial or segmental defects. The time interval between inserting a cement spacer and diagnosis of IMSO ranged from six months to nine years. Two cases were grade I, and one case each was grade III and IV. The clinical data are summarized in Table 1 and illustrated in Figs. 4, 5, 6 and 7.

**Table 1:** Summary of clinical data indicating the varying degrees of spontaneous osteogenesis

Case number	Age/Gender	Location	Causes of defect	Fixation	Grade of spontaneous osteogenesis	Time interval	Bone graft	Follow up
1	44 Yrs/F	Ulnar and radius	Severe fracture, infection	Plate	IV	6 Months	N	Clinically healed
2	37 Yrs/M	Tibia	Severe fracture, infection	Plate	I	9 Months	Y	Clinically healed
3	52 Yrs/M	Tibia	Infection	Nail	III	6 Months	Y	Clinically healed
4	29 Yrs/M	Tibia	Tumor resection	Brace	I	9 Yrs	N	New bone formation

## Discussion

The IM contains osteoinductive mediators including vascular endothelial growth factor (VEGF), transforming growth factor-β1 (TGF-β1), bone morphogenetic protein-2 (BMP-2), osteogenic cells including mesenchymal stem cells (MSCs), and adequate vascularity, and acts as an osteoconductive matrix (scaffold) [4, 7, 14–16]. Therefore, IM has osteogenic activity and potential for SO [4, 7, 8]. However, the osteogenic activity of the IM is often weak, and the SO is generally minimal and not constant, so a bone graft is necessary when using the classic IMT [16–20]. Our study showed that IMSO was not a rare event, especially in the animal experiment, but could be overlooked because it is not robust. Therefore, we proposed the concept of IMSO and found that the SO was caused by the enhancement of the osteogenesis activity of the IM due to various factors. Our study suggested that thebioactive bone tissue at the bone ends was the key factor involved in enhancing the osteogenic activity of the IM, followed by a concomitant inflammatory reaction.

### Bioactive bone tissue enhances the osteogenic activity of the IM

The IMs formed in ectopic locations (subcutaneous and intramuscular) did not have osteogenic potential and did not contain MSCs in the animal model studies reported by Catros et al. [21] and Pelissier et al. [22]. However, the IMs formed at the bone defects had osteogenic potential with higher concentrations of bone growth factors and cytokines, and contained MSCs [22, 23]. In this experimental study, the SO was most evident at the junction between the bone ends and the IM, similar to the results described by Klaue et al. [8], because the bone marrow and the residual osteotomy debris at the bone ends provided osteogenic cells or osteoprogenitor cells. In this study, case 1 (Fig. 4) and case 2 (Fig. 5) were partial bone defects, which also provided osteoprogenitor cells. These results suggested that the bioactive bone tissue at the bone ends contains osteoprogenitor cells or osteogenic cells for the SO, which was the main reason for the enhanced osteogenic activity of the IM.

### Local inflammatory reaction enhances the osteogenic activity of the IM

A local inflammatory reaction (infective or noninfective inflammation) can increase the release of inflammatory factors (mainly a series of cytokines) and promotes vascularization [24–26]. These inflammators include cytokines such as tumor necrosis factor-α(TNF-α), interleukin-1 (IL-1), and IL-6 that attract MSCs to the fracture site and are pro-osteogenic [24–26]. In addition, the upregulation of some inflammatory factors in the body fluids creates a receptive environment and accelerates bone formation, which was an important cause of rapid callus formation and hypertrophic ossification [23, 24]. Upregulated inflammatory factors are found in the serum and cerebrospinal fluid of patients with traumatic brain injury (TBI). Hotchen et al. [24] reported on a patient with femoral fractures and bone defects concomitant with a traumatic brain injury (TBI), in whom a bridging hard callus formed 48 days after the first-stage of IMT was performed. The rapid callus formation in this patient was attributed to the dual effects of TBI and IM. Clinical experience has revealed that a local infection with low toxicity can accelerate osteogenesis in fractures. Case 4 (Fig. 7) of this study involved infected bone defects, and case 1 (Fig. 4) and case 2 (Fig. 5) exhibited partial bone defects associated with infection. These results suggested that a concomitant local inflammatory reaction (aseptic inflammation and low toxic infectious inflammation) around the spacer during the formation of the IM may be another main reason for the enhanced osteogenic activity of the IM.

### Adequate time is a key factor for IMSO

In case 3 (Fig. 6) in this study, the SO was observed because the time interval between insertion of the PMMA and detection of new bone formation was long (nine years). In the other three cases, the time interval was six months or longer. In the animal experiment, the SO was only observed in rats that received the IMT for 12 weeks. The SO was not apparent at 6–8 weeks. Thus, the longer the PMMA remained in place, the more evident the SO. These results suggested that adequate time is an essential factor or condition for IMSO.

### Influence factors of IMSO

Varying degrees of IMSO were related to the quantity of osteogenic cells, the degree of concomitant inflammation, and the stability of the bone defects. IMSO was more obvious in animal experiments than in patients (100% vs. 4.1%). Because the clinical application of IMT is mostly for patients with infectious and open bone defects [18, 19, 26], whose periosteum at the bone end is stripped and the medullary cavity is washed, resulting in a clean residual end, so the osteogenic activity of the bone end is weak; In addition, in clinical practice, we often undertake intracorporeal formation of a spacer using the IMT or the bone cement spacer is too large or too small, which is easy to block the bone marrow overflow [18, 19, 20], affecting IMSO. Therefore, IMSO is rare clinically, but common under experimental conditions.

### Osteogenesis mode and grading of IMSO

Gruber et al. [26] observed the formation of isolated bone and cartilage foci within the IM. Our animal experiment showed that in addition to new bone formation, cancellous bone, nature bone and cartilage were found in the new bone; cartilage was located on the outer side while cancellous bone and nature bone were located on the inner side. These results indicated that the osteogenesis mode of IMSO might be endochondral osteogenesis. We divided the SO into four grades according to the amount and characteristics of the new bone formation. Grade I exhibited the formation of a bone precursor (cartilage), which is usually not visible on radiographs, but only a thickened IM as determined by the naked eye. Grades II-IV refer to different degrees of new bone formation that are visible on radiographs. In case 2, seen in Fig. 5, faint new bone formation was observed on radiographs indicating that the bone precursor (cartilage) was transforming into bone.

### The limitations and strengths of this study

This study has some limitations. This paper only reveals the IMSO phenomenon, and its detailed mechanism, intramembranous ossification or endochondral ossification, or osteoinductive osteogenesis, is not involved, and needs additional study.

Based on the hypothesis that the bioactive bone tissue may enhance the osteogenic activity of IM and lead to SO, a small amount of bone marrow implanted at the bone ends close to the bone cement spacer will enhance the bone activity of IM and lead to SO, which can promote the repair of the bone defect. Therefore, the IMSO phenomenon provides the possibility of using improved IMT to reconstruct bone defects in one stage.

## Conclusions

Varying degrees of SO confirm the existence of the IMSO phenomenon. Bioactive bone tissue or local inflammation and a long time interval are the primary reasons underlying enhancement of the osteogenic activity of IM and leading to SO, which tends to take place as endochondral osteogenesis.

## Data Availability

Data will be available by Qudong Yin upon request.

## Abbreviations

IM: Induced membrane
IMT: Induced membrane technique
SO: Spontaneous osteogenesis
PMMA: Polymethylmethacrylate
MSC: Mesenchymal stem cell
TBI: Traumatic brain injury

## References

[1] Masquelet AC (2020). The induced membrane technique. Orthop Traumatol Surg Res.

[2] Mathieu L, Tossou-Odjo L, L’Escalopier ND, Demoures T, Masquelet AC (2020). Induced membrane technique with sequential internal fixation: use of a reinforced spacer for reconstruction of infected bone defects. Int Orthop.

[3] Siboni R, Joseph E, Blasco L, Barbe C, Bajolet O, Diallo S, Ohl X (2018). Management of septic non-union of the tibia by the induced membrane technique. What factors could improve results?. Orthop Traumatol Surg Res.

[4] Yin Q, Sun Z, Gu S (2013). Progress of Masquelet technique to repair bone defect. Chin J reparative Reconstr Surg.

[5] Nau C, Simon S, Schaible A, Seebach C, Schröder K, Marzi I, Henrich D (2018). Influence of the induced membrane filled with syngeneic bone and regenerative cells on bone healing in a critical size defect model of the rat’s femur. Injury.

[6] Gessmann J, Rosteius T, Baecker H (2022). Is the bioactivity of induced membranes time dependent?. Eur J Trauma Emerg Surg.

[7] Han WF, Shen J, Wu HR, Yu SP (2017). Induced membrane technique: advances in the management of bone defects. Int J Surg.

[8] Klaue K, Knothe U, Anton C (2009). Bone regeneration in long-bone defects: tissue compartmentalisation? In vivo study on bone defects in sheep. Injury.

[9] Kanczler JM, Ginty PJ, White L (2010). The effect of the delivery of vascular endothelial growth factor and bone morphogenic protein-2 to osteoprogenitor cell populations on bone formation. Biomaterials.

[10] Zhang W, Zhu C, Wu Y (2014). VEGF and BMP-2 promote bone regeneration by facilitating bone marrow stem cell homing and differentiation. Eur Cells Mater.

[11] Nyman R, Magnusson M, Sennerby L, Nyman S, Lundgren D (1995). Membrane-guided bone regeneration. Segmental radius defects studied in the rabbit. Acta Orthop Scand.

[12] Soldatos NK, Stylianou P, Koidou VP, Angelov N, Yukna R, Romanos GE (2017). Limitations and options using resorbable versus nonresorbable membranes for successful guided bone regeneration. Quintessence Int.

[13] Allan B, Ruan R, Landao-Bassonga E, Gillman N, Wang T, Gao J, Ruan Y, Xu Y, Lee C, Goonewardene M, Zheng M (2021). Collagen membrane for guided bone regeneration in Dental and Orthopedic Applications. Tissue Eng Part A.

[14] Toth Z, Roi M, Evans E, Watson JT, Nicolaou D, McBride-Gagyi S (2019). Masquelet technique: effects of spacer material and micro-topography on factor expression and bone regeneration. Ann Biomed Eng.

[15] Christou C, Oliver RA, Yu Y, Walsh WR (2014). The Masquelet technique for membrane induction and the healing of ovine critical sized segmental defects. PLoS ONE.

[16] Kombate NK, Walla A, Ayouba G, Bakriga BM, Dellanh YY, Abalo AG, Dossim AM (2017). Reconstruction of traumatic bone loss using the induced membrane technique: preliminary results about 11 cases. J Orthop.

[17] Alford AI, Nicolaou D, Hake M, McBride-Gagyi S (2021). Masquelet’s induced membrane technique: review of current concepts and future directions. J Orthop Res.

[18] Wang JB, Yin QD, Gu SJ (2019). Induced membrane technique in the treatment of infectious bone defect: a clinical analysis. Orthop Traumatol Surg Res.

[19] Kang Y, Wu YW, Ma YH (2020). Primary free-flap tibial open fracture reconstruction with the Masquelet technique” and internal fixation. Injury.

[20] Wang P, Wu YW, Rui YJ, Wang JB, Liu J, Ma YH (2021). Masquelet technique for reconstructing bone defects in open lower limb fracture: analysis of the relationship between bone defect and bone graft. Injury.

[21] Catros S, Zwetyenga N, Bareille R (2009). Subcutaneous-induced membranes have no osteoinductive effect on macroporous HA-TCP in vivo. J Orthop Res.

[22] Pelissier P, Lefevre Y, Delmond S (2009). Influences of induced membranes on heterotopic bone formation within an osteo-inductive complex. Experimental study in rabbits. Ann Chir Plast Esthet.

[23] Cuthbert RJ, Churchman SM, Tan HB, McGonagle D, Jones E, Giannoudis PV (2013). Induced periosteum a complex cellular scaffold for the treatment of large bone defects. Bone.

[24] Hotchen AJ, Barr LV, Krkovic M (2018). Bridging hard callus at 48 days in an open femoral shaft fracture with segmental defect treated with a first-stage Masquelet technique: I wasn’t expecting that. Strategies Trauma Limb Reconstr.

[25] Huang H, Cheng WX, Hu YP, Chen JH, Zheng ZT, Zhang P (2017). Relationship between heterotopic ossification and traumatic brain injury: why severe traumatic brain injury increases the risk of heterotopic ossification. J Orthop Translation.

[26] Gruber HE, Gettys FK, Montijo HE (2013). Genomewide molecular and biologic characterization of biomembrane formation adjacent to a methacrylate spacer in the rat femoral segmental defect model. J Orthop Trauma.

