# Correction: Varying degrees of spontaneous osteogenesis of Masquelet’s induced membrane: experimental and clinical observations

**DOI:** 10.1186/s12891-023-06740-z

**Published:** 2023-07-27

**Authors:** Qudong Yin, Xueming Chen, Beichen Dai, Jun Liu, Ying Yang, Sheng Song, Yanping Ding

**Affiliations:** 1 Department of Orthopaedics, Wuxi Ninth People’s Hospital Affiliated to Soochow University, Wuxi, 214062 Jiangsu China; 2 Department of Radiology, Wuxi Ninth People’s Hospital Affiliated to Soochow University, Wuxi, 214062 Jiangsu China

**Correction:** ***BMC Musculoskelet Disord*** **24** **, 384 (2023)**

10.1186/s12891-023-06498-4

Following publication of the original article [1], the authors would like to remove Qudong Yin as co-corresponding author.

The author group has been updated above and the original article [1] has been corrected.
